# Riding on the Rails

**Published:** 1881-06

**Authors:** Luke Sharp


### "RIDING ON THE RAILS."

[Detroit Free Press.]

Why is it that men get so utterly selfish the moment they get into a railroad car? See how the lordly creature spreads himself. He turns over a seat, places himself comfortably in one corner, some newspapers beside him, puts his feet on the opposite corner, and the valise beside his feet, four seats taken up, while only one fare has been paid.

I do like to stir this selfish animal up.

I saw him, thus ensconced in a crowded Great Western Railroad car on the evening train that was to leave in a few moments for the all-night ride to the "Bridge." He was comfortable and his hat was down over his eyes so that he could see no hints to the effect that a seat was wanted, and so he could pretend he was asleep.

A woman with a fretful child in her arms looked wistfully at the seats, but passed on, as the occupant faintly snored. She went into the next car, which was even more crowded.

I was looking for a friend who was going out on that train, had found him, shook hands, and was passing back through the cars when I came across this sleeping beauty. It was too good a chance to be missed.

"Can I have this seat, sir?"

No answer. He was asleep.

"Wouldn't you allow me to sit here, please?

The gentle snoring continued.

I put the valise under the seat and sat down in its place.

"What are you doing with that valise?"

"Oh," said I, regretfully, "I am sorry to awaken you, I merely wished to occupy this seat."

"I guess you will find plenty of other seats in the car," answered he, gruffly, taking up the valise, placing it beside him and resting partly on it, with his feet still on the opposite seat, betaking himself to repose, now occupying only three seats.

The woman with the fretful baby comes by.

"Madam, will you take this seat?"

"Oh, I am very much obliged, but I'm afraid you will have to stand up, sir."

"Not at all," and so she gratefully sits down.

You see I have all the gratitude, while the other fellow might just as well have had it.

I again turn my attention to him.

"Would you kindly put down your feet, sir. I wish to sit here."

"Who in the —— why couldn't you stay where you were. Seems to me you're mighty officious."

"No doubt, but would you please remove those feet. Ah, thank you, sir, you see the car is very crowded."

"Oh go to ——."

We will assume that he said "The Bridge."

Another woman with a basket is gazing helplessly around and she looks as if she might help my neighbor with the fretful baby, if the poor little thing gets cross during the long night journey, so I say :—"Would you like this seat, ma'am ?"

I get ever so much more thanks, and am looked on by those around as a sort of philanthropist, while the woman with the basket squeezes in past the sleeping man, and sits down where his feet were.

"Is the little baby sick, ma'am ?" says the basket woman.

"No. She's frightened at the noise, and is not used with being out at night."

There, I knew these women would strike up a friendship on the basis of that baby, in an instant.

"I am afraid I shall have to trouble you to move that valise, sir."

"The valise is all right, where it is."

"I would very much like to sit there."

"Well, you can't ; these seats are paid for."

"Both of them?"

"Yes, both."

I know that this is not true, but I can't make him show his tickets.

"I will wait here till the conductor comes, and if you have the right to the two seats, all right."

By this time he was sitting bolt upright, and was very mad. I seized the opportunity to seize the valise and place it on the floor, sitting down beside him.

Now he *is* mad.

"Put that valise where it was," he demands, thoroughly awake.

"I will, the moment you show me your other ticket."

"Put that valise back, or I'll chuck you out of this car if it costs me $100." And the trouble was he was quite able to do it. The two frightened women opposite both offered to give up their seats, as the angry man cried:

"Now, for the last time, I tell you, to put that valise where you found it."

At this moment a big burly lumberman from the Saginaw region going home to Canada came down the passage. "Will you take my seat?" I asked.

"Oh, bless you, no; I'll sit here on the wood-box, I don't ——"

"That's all right, I am not going with this train and you are quite welcome to the seat."

And with hearty thanks the broad shouldered lumberman crushed down in my place, jamming my valise friend into rather limited space.

The train began to move.

"Good bye, Chawley," said I hurriedly. "This is my depot, I get off here and I beg of you, as a special favor, not to chuck out my friend, the lumberman." He didn't. LUKE SHARP.

### THE FIRST BOOK EVER PRINTED.

The first book ever printed was the Bible. It was printed at Metz between the inventor of the art, and Faust, who furnished the funds. For a long time after it had been printed and offered for sale, no person save the artists themselves knew how it had been accomplished. The work was so astonishing and the manner of its production so mysterious that the printers were believed by the ignorant to be in league with the evil one. The Bible was in two folio volumes, and contained 1282 pages. The American *Newspaper Reporter* says this first edition has been justly praised for the strength and beauty of the paper, the exactness of the register and the lustre of the ink. There are eighteen copies of it now in existence. The only copy in the United States was owned by James Lennox, Esq., of New York City, and cost $2,200.

